# Cross-Linked Composite Solid Polymer Electrolyte Doped with Li_6.4_La_3_Zr_1.4_Ta_0.6_O_12_ for High Voltage Lithium Metal Batteries

**DOI:** 10.1021/acsami.4c08181

**Published:** 2024-08-19

**Authors:** Lamartine Meda, Kutemwa Masafwa, Ayssia N. Crockem, Jere A. Williams, Nila A. Beamon, Jada I. Adams, Jeremiah V. Tunis, Lingyu Yang, Jennifer L. Schaefer, James J. Wu

**Affiliations:** † Department of Chemistry, Xavier University of Louisiana, 1 Drexel Drive, New Orleans, Louisiana 70125, United States; ‡ Deptartment of Chemical & Biomolecular Engineering, University of Notre Dame, Notre Dame, Indiana 46556, United States; § NASA Glenn Research Center, Cleveland, Ohio 44135, United States

**Keywords:** composite solid polymer electrolytes, solid-state battery, solid-state electrolyte, lithium metal battery, lithium-ion conductivity

## Abstract

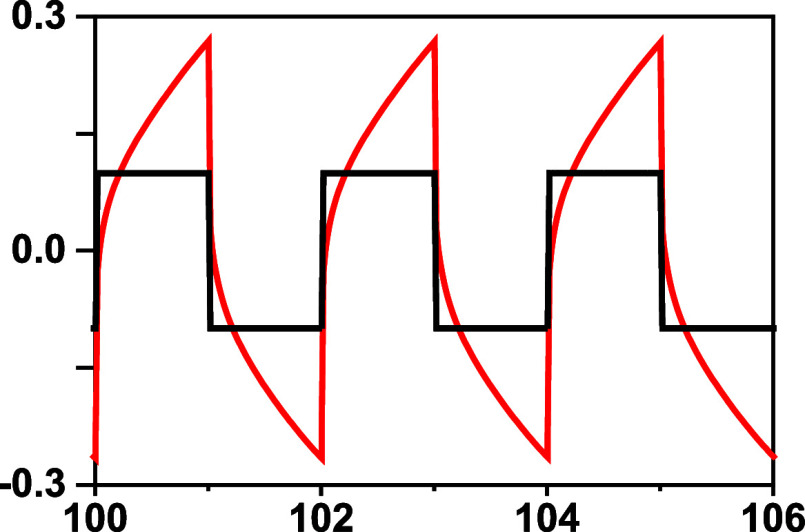

Composite solid polymer electrolytes (CSPEs) are safer alternatives to liquid electrolytes and excellent candidates for high-voltage solid-state batteries. However, interfacial instabilities between the electrodes and CSPEs are one of the bottlenecks in pursuing these systems. In this study, a cross-linked CSPE was synthesized based on polypropylene carbonate, polyethylene glycol methyl ether acrylate, polyethylene glycol diacrylate with additives including lithium bis(trifluoromethane)sulfonimide salt, and tantalum-doped lithium lanthanum zirconium oxide (LLZTO). Mass fractions of 10, 20, and 40% LLZTO were added to the CSPE matrix. In a symmetric cell, lithium plating and stripping revealed that the interface between the lithium metal anode and CSPE with 10% of the LLZTO (CSPE-10LLZTO) shows the most stable interface. The CSPE-10LLZTO sample demonstrated high flexibility and showed no degradation over 800 h of cycling at varying current densities. The ionic conductivity for the CSPE-10LLZTO sample at 40 °C was 6.4 × 10^–4^ S/cm. An all-solid-state full cell was fabricated with LiNi_0.5_Mn_0.3_Co_0.2_O_2_ as the cathode, CSPE-10LLZTO as the electrolyte and separator, and Li metal as the anode, delivering approximately 140 mAh/g of capacity. Differential scanning calorimetry measurements on CSPE-*x*LLZTO showed high miscibility and the elimination of crystallinity. Raman spectroscopy revealed uniformity in the structure. These findings demonstrate the capability of the CSPEs to develop high-voltage solid-state lithium metal batteries.

## 1 Introduction

Solid-state lithium metal batteries have been investigated as a replacement for Li-ion batteries (LIBs) due to the demands for safe (nonflammable and nonvolatile organic solvents) and high-energy storage for electric vehicles and other portable devices. To go beyond LIBs, the Li metal anode with a theoretical capacity of 3,860 mAh/g, which is approximately 10 times higher than the state-of-the-art graphite (372 mAh/g) anode, is very promising.^1,2^ The commercialization of rechargeable Li metal batteries has been immensely challenging due in part to the complex chemistry of Li metal.^3^ In a commercial battery, microporous separators allow for the transport of lithium ions within a liquid electrolyte back and forth between the anode and the cathode of a battery; however, these separators are vulnerable to the penetration of Li dendrites. This lithium penetration process can lead to an internal short circuit and severely limits the use of the Li metal anode. Substituting the flammable liquid electrolytes with solid electrolytes offers several advantages, such as more efficient cell packing and better thermal and mechanical stability.^4,5^ Exothermic reactions at the electrolyte/electrode interface and interphase are known to contribute to thermal runaway. The relatively very slow diffusion of organic material from the bulk of a polymer electrolyte to the interface, in comparison to a liquid electrolyte, is a reason for enhanced safety of polymer-based batteries in comparison to liquid-based batteries, even when the polymer is combustible.

Today, one of the most investigated and promising types of solid electrolytes is composite solid polymer electrolytes (CSPEs) made of poly(ethylene oxide) (PEO), lithium salt, and nanocomposite ceramic fillers.^6^ The most used ceramic filler is lithium lanthanum zirconium tantalum oxide (Li_6.4_La_3_Zr_1.4_Ta_0.6_O_12_, LLZTO), which is stable against Li metal and has a high ionic conductivity at room temperature (10^–4^–10^–3^ S/cm).^7,8^ This combination of LLZTO and PEO has become one of the most studied types of CSPE.^9−24^

Polymer electrolytes based on polycarbonates, including polypropylene carbonate (PPC), as used here, are known to have higher oxidative stability than the most common polymer electrolytes, such as PEO.^25^ Higher oxidative stability has also been observed for block copolymer electrolytes that contain blocks of polycarbonate and PEO blocks.^26^ PPC is also a widely available polymer in the commercial market. However, pure PPC-based electrolytes have less than desirable ionic conductivity and weak mechanical properties.

Here, we overcome these challenges by creating a cross-linked, interpenetrating network polymer electrolyte by polymerizing a comb monomer polyethylene glycol methyl ether acrylate (PEGMEA) and cross-linking monomer polyethylene glycol diacrylate (PEGDA) in the presence of PPC. The use of both the comb monomer and the cross-linking monomer allows for enhanced ionic conductivity above that of the cross-linking monomer alone, while the cross-linking monomer enables the creation of a freestanding polymer electrolyte film, and both of these monomers are also widely available.^27^ Cross-linking can significantly improve the properties of these polymers, such as giving them a rubber-like characteristic, which can improve the contact between the electrodes and polymer electrolytes. Interfacial resistance in CSPEs is one of the major drawbacks in lithium-ion transport, and therefore, understanding the Li metal/electrolyte interface is of paramount importance, which can lead to improving the performance of solid-state lithium metal batteries.^28^

In this article, we report on the bulk and interfacial properties of this cross-linked composite solid polymer electrolyte. Lithium bis(trifluoromethanesulfonyl)imide (LiTFSI) was used as the salt due to its interfacial stability against Li metal. The ionic conductivity improved when 10, 20, and 40% mass fractions of LLZTO were added. A representative sample of 10% LLZTO added to the CSPE matrix (CSPE-10LLZTO) demonstrated stable cycling over 800 h. An all-solid-state full cell made of LiNi_0.5_Mn_0.3_Co_0.2_O_2_ (NMC), CSPE-10LLZTO, and Li metal anode showed that the solid electrolyte performed very well.

## 2 Experimental Section

### 2.1 Preparation of CSPE-*x*LLZTO

The CSPE-*x*LLZTO synthesis was based on the semi-interpenetrating polymer network structure, which has been described previously.^29,30^ A brief description is given here. Unless otherwise specified, all reagents were purchased from Sigma-Aldrich and the experiments were performed in an argon-filled glovebox with a controlled environment. Samples were prepared in two different laboratories. The cross-linked polymer matrix (CSPE-0LLZTO) was first prepared by dissolving a known quantity of PPC, PEGMEA, PEGDA, and LiTFSI in acetone along with 2 wt % of the free radical thermal initiator 2,2′-azobis(2-methylpropionitrile) (AIBN). A series of CSPE-*x*LLZTO samples were then prepared by adding 10, 20, and 40% by mass of LLZTO (MSE Supplies, particle size 400–600 nm) to the prepolymer solution. The solutions containing LLZTO were sonicated in icy water for 2 h to disperse the particles. In the laboratory at the Xavier University of Louisiana, casting was performed by pouring the solutions onto a polytetrafluoroethylene dish and leaving it to allow for solvent evaporation for 48 h in the MBraun glovebox. The composite was then cross-linked by heating at 80 °C under vacuum for 1 h before peeling and being punched into a 16 mm diameter disk. In the laboratory at the University of Notre Dame, the films were cast in an ambient atmosphere, and the bulk of the acetone was evaporated in the hood. Then, the samples were transferred to an LC Technologies argon glovebox equipped with a built-in vacuum oven. The samples were placed into a vacuum oven and pumped down. Then, while holding vacuum, the temperature in the oven was ramped up to 80 °C over a period of 90 min. The samples were maintained under vacuum in the oven at 80 °C for an additional 12 h before being moved to storage under ambient pressure in the argon glovebox. Samples prepared via either procedure were found to have equivalent ionic conductivity and glass transition temperature.

### 2.2 Material Characterizations

The crystal structure and purity of the LLZTO were characterized using powder X-ray diffraction (XRD) in the Bragg–Brentano geometry from 10° to 80° in 2-Theta in a Rigaku MiniFlex benchtop with Cu K_α_ radiation (λ = 1.5418 Å). Additionally, the CSPE-*x*LLZTO samples were examined by XRD as well.

The samples’ morphology and surface characterization were performed in a Hitachi S-3400 scanning electron microscope equipped with energy-dispersive X-ray spectroscopy (EDS) and elemental mapping. First, the samples were mounted onto a scanning electron microscope holder inside the glovebox and placed in a sealed container to prevent exposure to air and moisture. Then, the holder was carefully transferred under inert gas to the SEM chamber for the analysis of the sample.

Differential scanning calorimetry (DSC) measurements were conducted using a TA Instruments Q2000. Polymeric samples of at least 5 mg each were crimped in aluminum DSC sample pans. The DSC scans were run between −80 and 140 °C at a scan rate of 10 °C/min under a nitrogen flow of 25 mL/min. The temperature was decreased to −80 °C and then increased to 140 °C, and this cycle was repeated. The second cooling scan was used to calculate the samples’ glass transition temperature (*T*_g_).

Raman spectroscopic studies were performed on an NRS-5100 confocal micro-Raman spectrometer from Jasco with an excitation wavelength of 532 nm. The samples were sandwiched between one quartz microscopic slide and one glass coverslip. Carbon tape was used to seal the edges of the glass to minimize the sample’s exposure to moisture. The following were the settings of the device: 20× magnification, 50 × 1000 μm slit dimension, and 4000 μm aperture. The samples were exposed to the laser for 30 s for one accumulation each in the 80–2000 cm^–1^ range.

Linear sweep voltammetry asymmetric cell testing (SS|CSPE-10LLZTO|Li) was performed to establish the electrochemical window of the cross-linked polymer electrolyte in the range of 0 to 6 V at a scan rate of 2 mV/s.

### 2.3 Electrochemical Characterizations

#### 2.3.1 Ionic Conductivity

All the samples’ ionic conductivity (σ, S/cm) was calculated from the electrochemical impedance spectroscopy (EIS) data in the 25–85 °C temperature range. Before each measurement, the temperature was held constant for approximately 60 min. Coin cell types CR2032 were assembled by inserting a CSPE-*x*LLZTO film (∼230 μm thick) between two stainless steel blocking electrodes (SS|CSPE-*x*LLZTO|SS) and pressing the material with a spring. The EIS for the cells was carried out in the frequency range of 800 kHz–0.1 Hz using a Gamry 1000 Interface potentiostat, and the data were displayed as Nyquist plots.

#### 2.3.2 Interfacial Stability

All samples’ overall resistance (*R*_overall_) was measured at 40 °C at an open-circuit voltage (OCV) by monitoring the EIS response over time of a symmetric cell with the potentiostat in the galvanostatic mode. This temperature was chosen because it falls in the working temperature range of solid polymer electrolytes and the ionic conductivity is relatively high. The CSPE-10LLZTO was selected for further evaluation because it showed the best overall interfacial stability. The CSPE-0LLZTO was tested as a control for comparison. The sample was assessed by lithium plating and stripping for 1 h each at current densities ranging from 50 to 400 μA/cm^2^.

#### 2.3.3 All-Solid-State Cell

The effectiveness of the solid electrolyte was tested in a full cell using NMC (Ni:Mn:Co = 5:3:2, MTI Corp.) as the active cathode, CSPE-10LLZTO as the separator and electrolyte, and Li metal as the anode. The cathode was prepared by mixing NMC, Super-P carbon black (Alfa Aesar) as a conductivity enhancer, and poly(vinylidene fluoride) (PVDF, average Nw ∼ 534,000, Aldrich) as the binder in the ratio 70:20:10 wt %, respectively. The powders were placed in a pestle, and 2 mL of 27.5 g/L PVDF/NMP (*N*-methyl-2-pyrrolidone, 99.5%, anhydrous, Sigma-Aldrich) was added. The solution was transferred to a vial and stirred overnight to yield a black slurry that was cast on 16 μm thick aluminum foil (MTI, Corp). The samples were dried in a vacuum oven (MTI) at 110 °C overnight and punched into 15 mm diameter disks. The loading mass was approximately 1.165 mg/cm^2^, and the weight was measured using a microbalance (Mettler Toledo).

Approximately 1.0 μL of 1.0 M lithium hexafluorophosphate (LiPF_6,_ LP30; EC: DMC = 50:50 v/v, Sigma-Aldrich, battery grade) in ethylene carbonate (EC) and dimethyl carbonate (DMC) was used on the cathode to improve ionic conduction. Coin cells were assembled by using an electric crimper inside the glovebox. The cells were cycled in a temperature-controlled oven (Arbin Instruments) at 40 °C in the 2.8–4.5 V voltage range versus Li/Li^+^ at a current density of 10 mA/g. Cyclic voltammetry (CV) was performed for the NMC cells in the same voltage range at a scan rate of 0.2 mV/s to evaluate the chemical nature of the redox reactions. Liquid cells were assembled using a Whatman inorganic separator to compare to solid-state cells.

### 2.4 Post-mortem Analysis

For the CSPE-10LLZTO sample, the cells were taken inside the glovebox for decrimping after lithium plating and stripping, and the electrodes and electrolytes were carefully separated whenever possible. Next, cross sections of CSPE-10LLZTO were prepared by cutting the sample with a surgical blade for SEM analysis. The effort to separate the lithium anode from the CSPE-0LLZTO was unsuccessful.

## 3 Results and Discussion

### 3.1 Physicochemical Properties

**Figure 1:**
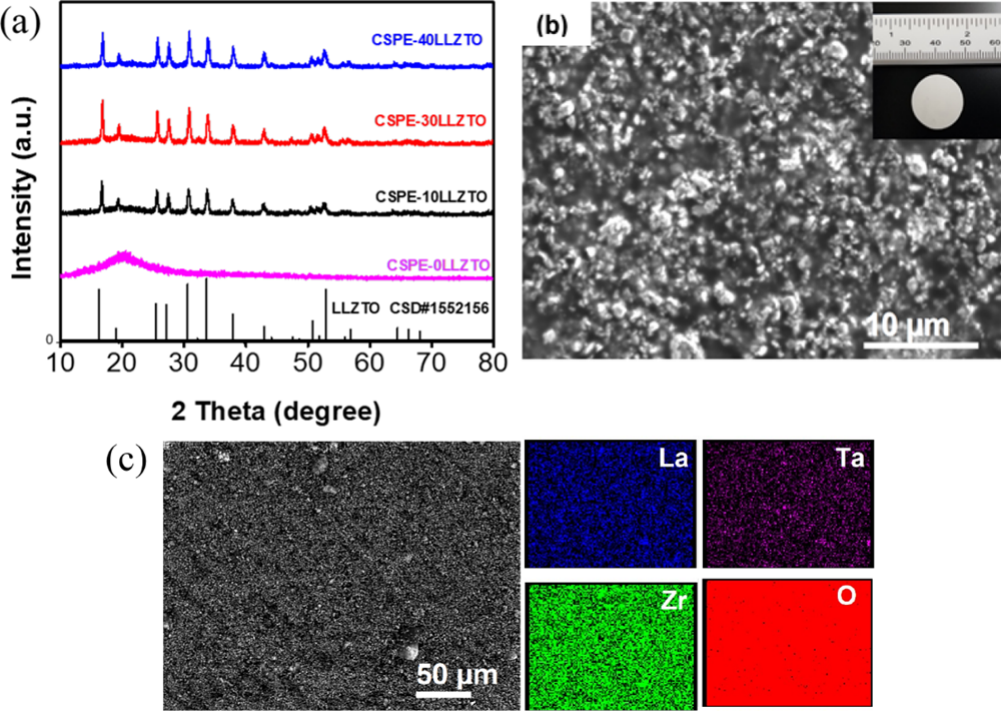
(a) XRD patterns of CSPE-*x*LLZTO (*x* = 0, 10, 20, and 40%); (b) representative SEM image of CSPE-10LLZTO (inset shows the optical image of the CSPE-10LLZTO); (c) SEM/EDS elemental mapping of CSPE-10LLZTO.

The crystallinity of the CSPE-*x*LLZTO and LLZTO powder samples was determined by powder XRD (Figure 1). The XRD pattern of the CSPE-0LLZTO shows a broad peak from 10° to 30° with no additional peaks present, indicating that the CSPE-0LLZTO is amorphous. The XRD patterns of the CSPE-*x*LLZTO were found to be identical to the cubic LLZTO reference pattern (CSD#1552156 or JCPDF-80-0457). Hence, the results suggested that the LLZTO is stable in the cross-linked polymer matrix. The LLZTO XRD pattern is shown in Figure S1.

The surface morphology and EDS elemental analysis of CSPE-10LLZTO, which is the sample chosen for further study, are shown in Figure 1b,c. The images showed that the LLZTO particles are well embedded in the solid polymer matrix, facilitating a continuous and homogeneous structure. EDS elemental mapping shows the overall distribution of La, Zr, Ta, and O in the CSPE-10LLZTO sample, indicating that LLZTO was well dispersed on the surface. A relatively small amount of agglomeration is noticeable on the SEM surface. A SEM picture of the LLZTO (400–600 nm) particles is shown in Figure S2.

### 3.2 Thermal Properties

DSC was used to evaluate the thermal properties of the electrolyte. In Figure S3, we observed the absence of melting point peaks in the DSC curves. This implies the suppression of crystallinity in the polymer matrix, which would increase the lithium-ion conductivity. The single *T*_g_ was the only transition observed in the DSC curves of the samples. This single *T*_g_ implies that the polymer matrix’s PPC and PEG segmental portions were homogeneously mixed with no phase segregation. The addition of ceramic LLZTO particles only slightly decreased the *T*_g_ of the polymer matrix, with the CSPE-20LLZTO recording the lowest *T*_g_. The observed *T*_g_ of the samples is tabulated in Table 1.

**Table 1:** Observed CSPE *T*_g_

sample	glass transition (*T*_g_)
0% LLZTO CSPE	–54.2 °C
10% LLZTO CSPE	–55.1 °C
20% LLZTO CSPE	–57.7 °C
40% LLZTO CSPE	–55.7 °C

### 3.3 Ionic Transport

**Figure 2:**
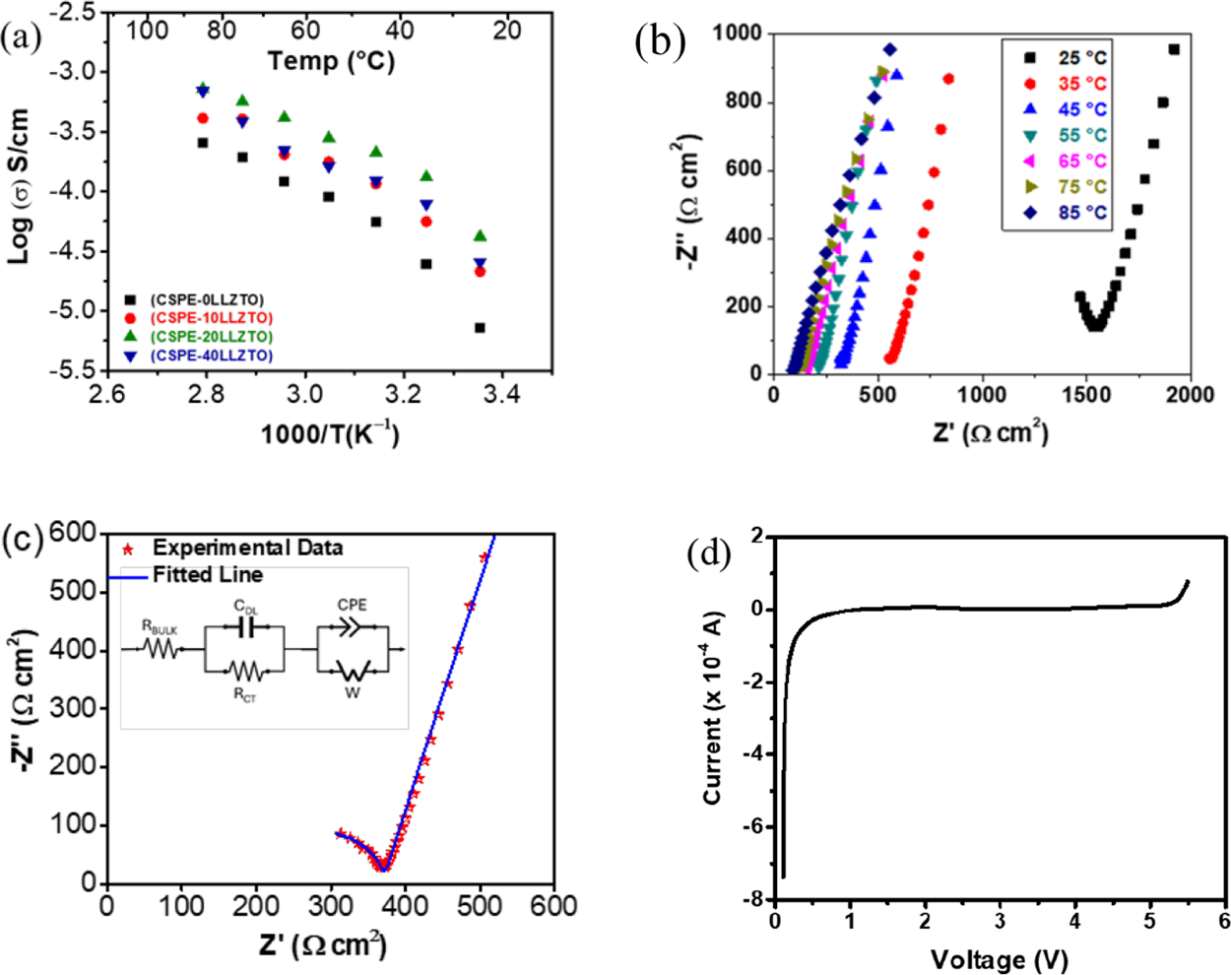
(a) Arrhenius plot shows the ionic conductivity as a function of the temperature of the CSPE-*x*LLZTO; (b) Nyquist plots of the CSPE-10LLZTO taken in the temperature ranging from 25 to 85 °C; (c) Nyquist plot to the CSPE-10LLZTO at 40 °C fitted using an equivalent circuit; (d) linear sweep voltammetry asymmetric cell testing (SS|CSPE-10LLZTO|Li) scanning from 0 to 6 V at a scan rate of 2 mV/s.

The ionic conductivity (Figure 2a) increased for all of the samples as the temperature increased. This is a typical trend in solid polymer electrolytes since higher temperatures enhance the movement of Li ions and polymer chains.^31^ As the LLZTO mass fraction increases from 10 to 20%, we notice that the ionic conductivity also increases; however, CSPE-40LLZTO shows that the ionic conductivity decreases. Typically, an increase in ionic conductivity in CSPEs has been attributed to varied mechanisms in the literature, including depression of the polymer glass transition and increase of polymer chain segmental motion, increase in the effective mobile ion concentration due to interaction between the anion and the particle surface, preferential ion transport pathways along the particle surfaces, and ion transport through both the inorganic and polymer matrices in the case of active Li^+^ conducting inorganics.^31^ The latter mechanism involves ion conduction in the inorganic phase that is inhibited due to high resistance to ion transport across the particle–polymer interface. The Arrhenius plots of the samples show a nonlinear (curvature) dependence of the ionic conductivity as a function of temperature. However, in the high-temperature range, a linear dependence can be extrapolated for the samples that contained LLZTO. Overall, the ionic conductivity behavior as a function of temperature follows the Vogel–Tamman–Fulcher (VTF) equation, indicating that the preferred conduction pathway is through the polymer matrix rather than the LLZTO. Although the ionic conductivity increased with an increase in LLZTO content to 20%, this increase also cannot be attributed solely to the decrease in the glass transition temperature for the CSPE-20LLZTO and increase in the polymer segmental mobility, as the changes in the glass transition temperatures were relatively small (Figure S3). Nevertheless, the *T*_g_ corroborated the ionic conductivity data, showing that the CSPE-20LLZTO sample has the highest conductivity and the lowest *T*_g_.

The CSPE-0LLZTO has a Li^+^ conductivity (σ) of 7.2 × 10^–6^ and 2.5 × 10^–4^ S/cm at 25 and 85 °C, respectively. The highest Li^+^ conductivity at 25 °C was achieved with the CSPE-20LLZTO, which was 4.1 × 10^–5^ S/cm; at 85 °C, the conductivity was 7.2 × 10^–4^ S/cm. The *R*_overall_, which was used in the calculation of the ionic conductivity, was obtained from the fitted spectra by an equivalent circuit that is made of a resistor *R*_bulk_ (current collector, electrolyte, and separator resistances) in series with *R*_ct_ (charge transfer resistance), in parallel with a capacitor *C*_dl_ (capacitance between particles or double layer capacitance), and in series with a constant phase element (CPE), which is used for nonideal capacitor behavior, and Warburg diffusion constant (*W*). Notice that in this model, *W* affects the kinetic and the diffusion which appears as a straight line. The following relationship was used to determine the ionic conductivity: *R*_overall_, *l* is the thickness of the electrolyte film in cm, and A is the electrolyte area in cm^2^.
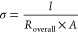


Adding the LLZTO ceramic filler increased the ionic conductivity by at least 1 order of magnitude. At 40% mass fraction of LLZTO, the ionic conductivity decreased, which is not surprising given the VTF model for CSPEs. The Nyquist plots for the CSPE-10LLZTO, taken between two stainless steel blocking electrodes, are shown in Figure 2b, and a simplified Randles model is shown in Figure 2c for the measurement taken at 40 °C. The presence of one semideveloped Nyquist plot suggests that the bulk electrolyte is a homogeneous mixture and a signature of a single dominant pathway for charge transport.^32^ The impedance graphs for CSPE-0LLZTO and CSPE-20LLZTO are shown in Figure S4.

### 3.4 Raman Spectroscopy

**Figure 3:**
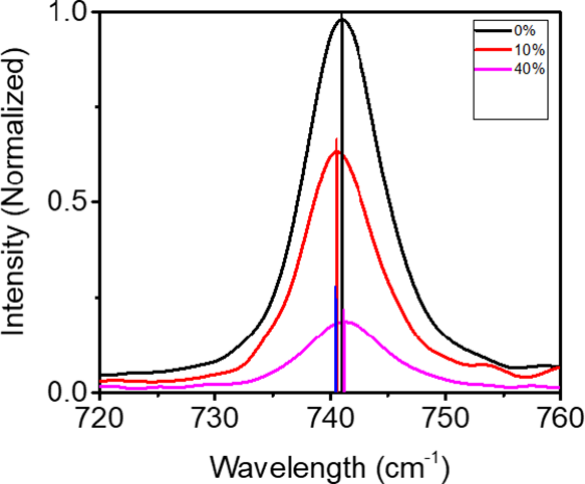
Raman graphs of CSPE-xLLZTO from 720 to 760 cm^–1^.

Raman spectroscopy measurements were performed on the CSPE-*x*LLZTO instrument to investigate the molecular interactions of the TFSI^–^ anion to determine if the particle–anion interactions are responsible for the increase in the ionic conductivity that is observed for the composite samples. Surprisingly, there is little shift in the peak at ∼740 cm^–1^ that is sensitive to the bending of the TFSI^–^ anion (Figure 3). This result implies that the TFSI^–^ anion does not bind to the LLZTO surface. Therefore, the increase in ionic conductivity must be attributed to another mechanism, such as substantial Li^+^ transport near the particle surfaces.

### 3.5 Interfacial Stability

**Figure 4:**
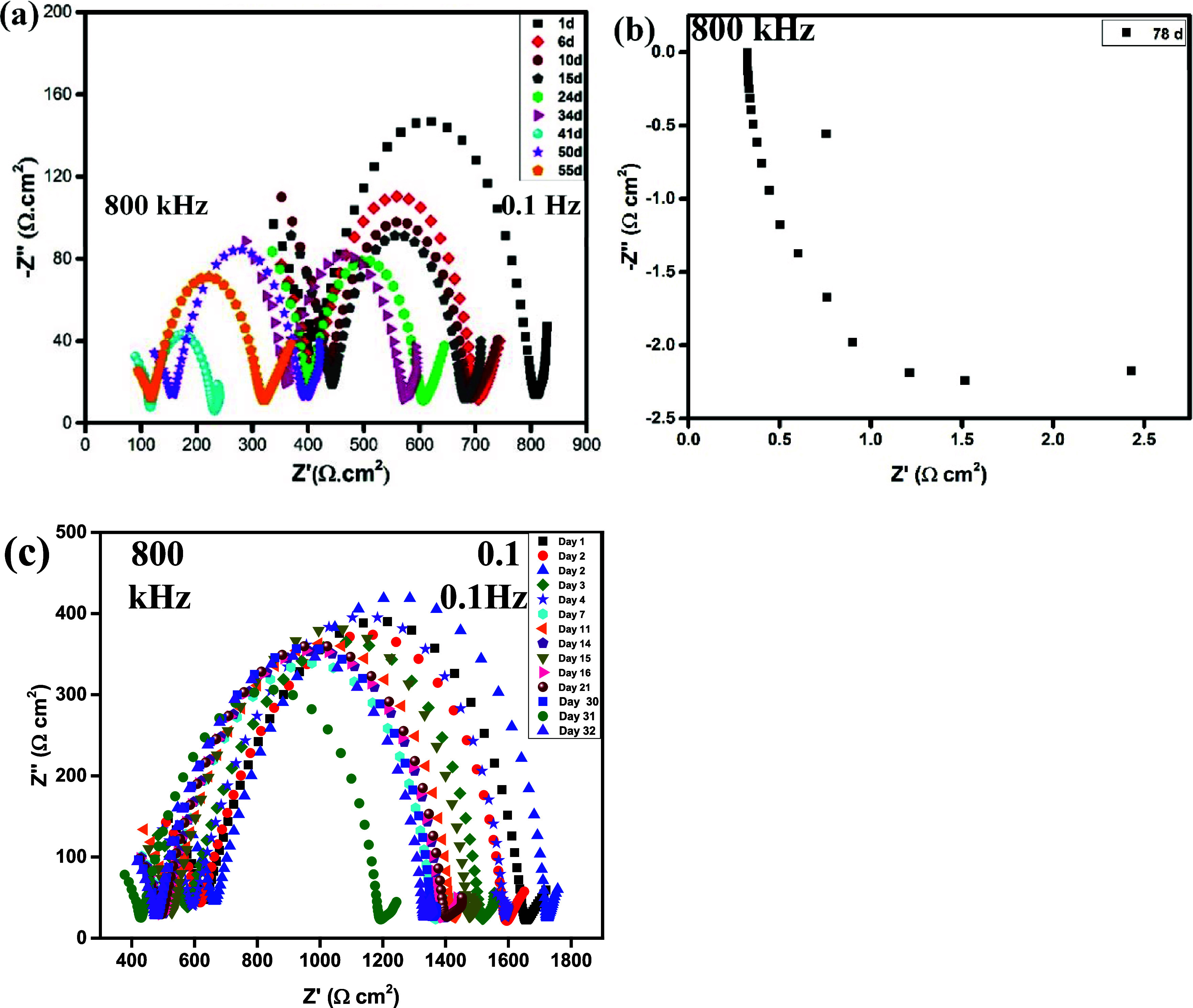
Representatives Nyquist plots taken at the open-circuit voltage at 40 °C in the frequency range from 800 kHz to 0.1 Hz; (a, b) Li|CSPE-10LLZTO|Li and (c) Li|CSPE-0LLZTO|Li.

The interfacial stability between the Li metal anode and CSPE-10LLZTO was evaluated by monitoring the AC-impedance response at 40 °C under OCV conditions. Figure 4 shows the Nyquist plots of the symmetrical cells Li|CSPE-10LLZTO|Li and Li|CSPE-0LLZTO|Li. To evaluate the Nyquist plots, it is divided into three frequency regions: (a) high frequency, (b) medium high frequency, and (c) low frequency. The high-frequency region is assigned to *R*_interface_, the medium high-frequency region is assigned to *R*_ct_, and the low-frequency region is assigned to the Warburg impedance (*W*) for the mass transfer. This is the common assignment used in the literature to deconvolute complex EIS responses.^33^*R*_overall_ represents the summation of these resistances in the cell. The values of resistance at different days at the OCV for the CSPE-0LLZTO and CSPE-10LLZTO with a thickness of ∼230 μm at 40 °C are shown in Table 2. The percentage represents a decrease from the original resistance.

**Table 2:** Values of Resistances at the Open Circuit Voltage for an Electrolyte with a Thickness of Approximately 230 μm before the Measurement was Taken at 40 °C

days	*R*_interface_ (Ω cm^2^) CSPE-0LLZTO	*R*_ct_ (Ω cm^2^) CSPE-0LLZTO	*R*_overall_ (Ω cm^2^) CSPE-0LLZTO	*R*_interface_ (Ω cm^2^) CSPE-10LLZTO	*R*_ct_ (Ω cm^2^) CSPE-10LLZTO	*R*_overall_ (Ω cm^2^) CSPE-10LLZTO
1	624	1050	1650	400	400	800
6	506 (37%)	844 (20%)	1350 (18%)	400 (0%)	300 (25%)	700 (12%)
10	520 (36%)	925 (12%)	1425 (14%)	425 (+6%)	275 (31%)	700 (12%)
15	500 (33%)	1000 (5%)	1500 (10%)	450 (+13%)	225 (44%)	675 (16%)
24	534 (45%)	650 (38%)	1400 (15%)	400 (0%)	200 (50%)	600 (25%)
34	432 (36%)	768 (64%)	1200 (23%)	350 (13%)	225 (44%)	575 (28%)
41				100 (75%)	125 (69%)	225 (72%)
50				150 (63%)	250 (38%)	400 (50%)
55				100 (75%)	225 (44%)	325 (41%)
78						0.5 (0.1%)

The Nyquist plot of the Li|CSPE-10LLZTO|Li cell (Figure 4a), on the first day of testing at the OCV, shows a *R*_overall_ of 800 Ω cm^2^ at 0.1 Hz, which was observed where the semicircle intersects the real axis (*Z*′). 50% of *R*_overall_ is *R*_ct_ (400 Ω cm^2^), and the other 50% is the *R*_interface_. After 15 days of testing, the *R*_interface_ increased by 13%; however, the *R*_ct_ decreased by 44%. After 34 days of storage under the same conditions, the *R*_overall_ decreased to 575 Ω cm^2^, which is a drop of 28%. The *R*_interface_ fell by 13%, but *R*_ct_ stayed constant. The *R*_ct_ is decreasing more rapidly than the *R*_interface._ We notice that despite the formation of the SEI, there was no increase in the *R*_overall_ of the CSPE-10LLZTO during the first month of storage time. It is normal for the *R*_interface_ to increase with time. However, the decrease in *R*_ct_ showed that the interface is still dynamic and wetting the electrode. After 41 days of testing, the *R*_overall_ dropped drastically from 28% (575 Ω cm^2^) to 72% (325 Ω cm^2^), while the *R*_ct_ and *R*_interface_ dropped by 69 and 75%, respectively. This precipitous decrease represents a soft short circuit (soft short) in the cell. After 55 days of testing, the *R*_overall_ increased by 44% (325 Ω cm^2^) from the last drop of 72% (225 Ω cm^2^) and the *R*_ct_ increased by 80%. However, the *R*_interface_ stayed the same, which implied that the interphase was completely nonexistent, and the electrolyte and lithium metal were completely wetted where the electrolyte behaved as a mixed conductor. The *R*_interface_ dynamically changes to lower resistances at both high and low impedances. That can cause electron leakage in some parts of the electrolyte. After 78 days of testing, the overall resistance at high frequency was 0.5 Ω cm^2^ (a 100% drop), and the shape of the graph completely changed (Figure 4b). The fact that the plot appeared at the negative quadrant at high frequency indicated the presence of inductance, which caused the cell’s electronic short circuit (hard-short) because of the growth of dendrites. A similar phenomenon has been observed in the literature with a symmetric ceramic cell made of Li|LLZTO|Li.^34^

For comparison, CSPE-0LLZTO was also tested and showed a tremendous decrease in *R*_overall_. We believe that the soft shorts are responsible for this acceleration in decreasing the *R*_overall_, although the cells continue to cycle for 55 days. Dendrites’ growth erroneously showed improved Li|CSPE-0LLZTO interface contact. The SEI layer decreased with time, and the *R*_overall_ of the cell also decreased with time. After 52 days, the *R*_overall_ value was approximately 200 Ω cm^2^, and the *R*_interface_ completely disappeared. All the resistances (*R*_interface_, *R*_ct_) were dynamic and continuously changed with time toward lower resistance. Typically, impedance increases upon the formation of the SEI to protect the electrode from failure. In our case, the drastic decrease in resistance of the Li|CSPE-0LLZTO implies that a soft short took place through either electron leakage of the electrolyte via dendrite growth or wetting of the electrode surface. We do not believe a hard short circuit happened where the electrolyte behaves as a resistor. Instead, a mixed conductor with electronic and ionic conductions is more likely to result, which is a phenomenon that is common with polymer electrolytes upon soft short-circuiting.^35,36^

Adding LLZTO beyond 10% did not further enhance the interfacial resistance, but there was an increase in ionic conductivity. A similar phenomenon has been observed in other architectures, where an increase in ionic conductivity does not necessarily lead to a stable interface. For example, in the Li/LLZTO/Li and Li/LLZT-2LiF/Li symmetrical cells at 25 °C, the Li/LLZT-2LiF/Li was found to have a lower interfacial resistance (345 Ω/cm^2^) compared to the Li/LLZT/Li (1260 Ω/cm^2^). However, the latter had a higher ionic conductivity.^37^

The EIS results showed that the addition of LLZTO improved the stability of the interface. Numerous factors could enhance the interfacial stability including the strength of the electrolyte and the thickness of an insulating layer. It has been reported that LLZO with the nominal composition of Li_7_La_3_Zr_2_O_12_ is stable against molten lithium and lithium metal^38−43^ and that stability could significantly improve the *R*_overall_. Indeed, a similar observation has been made in a Li/PEO-LLZTO/Li cell due to the gradual activation of the PEO/LLZTO electrolyte interface.^44^

**Figure 5:**
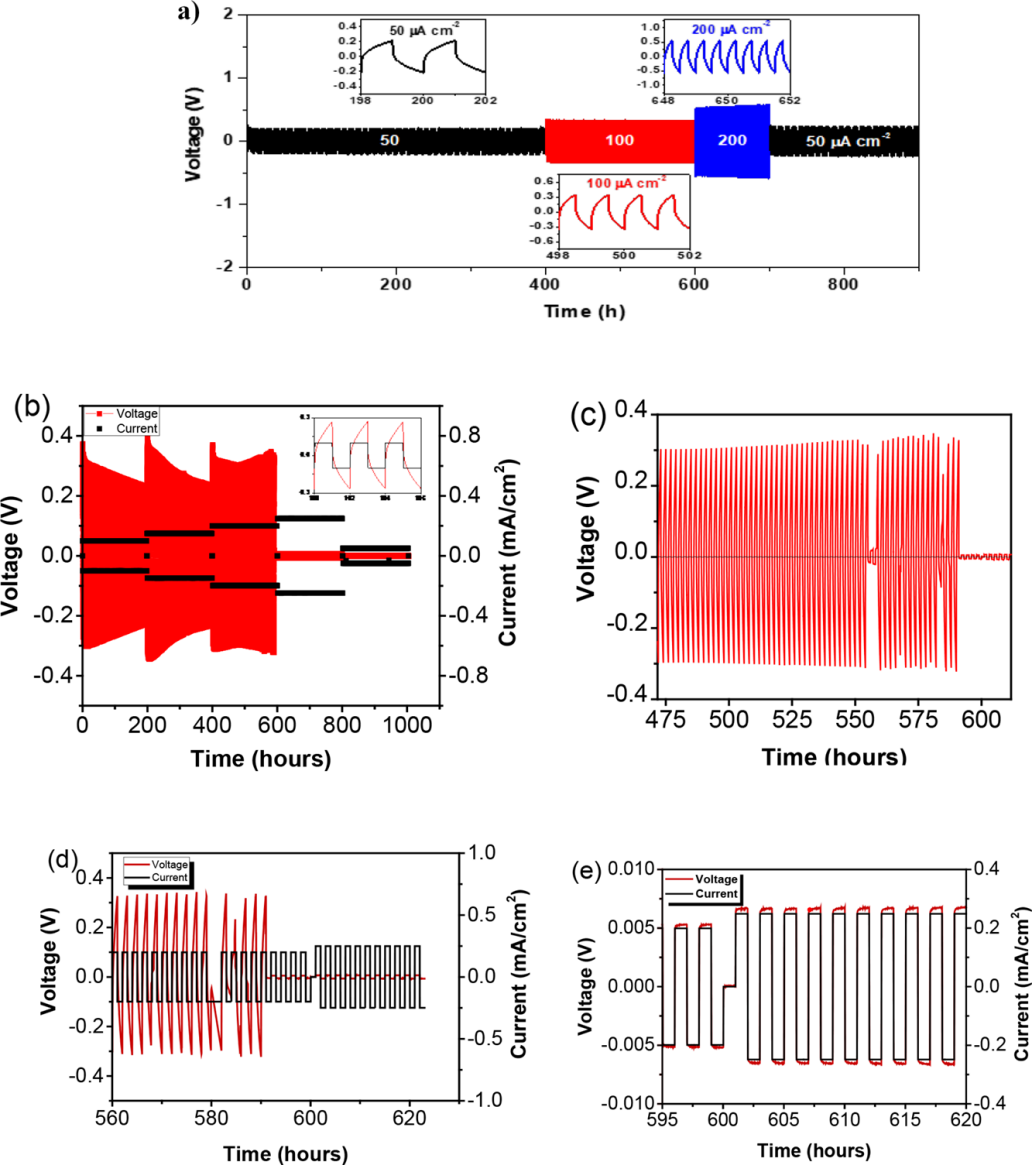
Li plating and stripping at 40 °C for (a) CSPE-10LLZTO from 50 to 200 μm/cm^2^; (b) CSPE-0LLZTO 50 to 200 μm/cm^2^; graphs are enlarged from (c) 475 to 600 h, (d) 560 to 620, showing soft short and hard short, and (e) 550 to 600 h showing the soft short.

To investigate the efficiency of the CSPE-10LLZTO in an electrochemical system, galvanostatic plating and stripping of Li metal in symmetrical cells was performed at 40 °C and constant current densities of *j* = 50, 100, and 200 μA/cm^2^. The overpotentials produced by kinetic complications were measured to determine the extent of the electrochemical reaction in the time intervals of 2 h, where the polarity was reversing every hour. Figure 5 shows the lithium plating and stripping in the Li|CSPE-10LLZTO|Li cell at 50 μA/cm^2^ for 200 h. The first cycle started at an overpotential of 0.25 V but decreased to a stable potential of ∼0.20 V and stayed constant. That decrease in the overpotential implies improvement in the Li|CSPE-10LLZTO interface. An increase in polarization to 0.35 V was observed when the rate was increased to 100 μA/cm^2^. The overpotential remained constant at 0.35 V for 200 h in this case. The current density was further increased to 200 μA/cm^2^; again, the overpotential increased and stabilized to 0.50 V for over 200 h. Finally, to check the retention memory of the electrolyte, the current density was reduced to its initial rate of 50 μA/cm^2^, and the overpotential returned to its original value and stayed stable for an additional 200 h. We observed no significant changes in the plating and stripping profiles at up to 200 μA/cm^2^. In case there was dendritic (short circuit) formation of Li metal, it would have been manifested by the appearance of a sharp polarization peak in the cycling process of Li symmetrical cell,^45^ which was not observed in the Li|CSPE-10LLZTO|Li for over 800 h. This result provided good evidence about the compatibility of Li metal with the CSPE-10LLZTO. Increasing the current density to 400 μA/cm^2^ showed a slight increase in overpotential for the CSPE-10LLZTO (Figure S5).

We also noticed that the shape of the voltage profile during charging is arching (Figure 5a, inset b) instead of a sharp peak. That may be because the current density is very low, since we observe no sharp peak when a ceramic separator (Whatman GF separator) is filled with liquid electrolyte (LP30, Sigma-Aldrich) in a symmetric cell during the first charge at 50 μA/cm^2^. A sharp peak due to SEI formation is typically observed for liquid electrolytes, but we notice that we are working at a much lower current density. However, even at a current density of 400 μA/cm^2^, we did not observe a sharp peak during the first charging but rather an arching of the voltage profile. That explains the early tortuous pathway of the lithium-ion in the polymer matrix at the very beginning of the cycling process compared to the liquid electrolyte, where the arching appears much later in the cycling process of the liquid electrolyte, although it is strongly dependent on the current density.

For comparison, lithium plating and stripping of CSPE-0LLZTO were also performed at 50 μA/cm^2^. For the first cycle, the overpotential was 0.55 V, which is higher than the CSPE-10LLZTO under the same conditions, and the voltage continuously decreased with time while the current was kept constant. Between 550 and 560 h (Figure 5c), the voltage dropped to ∼20 mV, indicating a soft short. However, after a few cycles, the overpotential returned to 0.3 V, and lithium plating and stripping continued. This type of phenomenon indicates that this cross-linked polymer matrix has self-healing properties. As the cell continued to cycle for an additional 30 h, the voltage dropped again, and this time to 5 mV at the same current density of 200 μA/cm^2^, and the voltage profile was quite different. A flat plateau that makes the voltage profile look like a rectangle is observed (Figure 5d,e). The extremely low overpotential and the rectangular shape where the voltage and the current are at the same height also represent soft short. To check that the cell was indeed soft and short, EIS was carried out under the OCV conditions. As demonstrated in Figure S5, a spectrum was obtained showing an intercept at low frequency (0.1 Hz) of the real axis at 160 Ω/cm^2^; however, the *R*_interface_ is approaching zero resistance. The fact that a spectrum is observed represents ionic conduction. Therefore, the electrolyte behaves as a mixed conductor with localized soft shorts.

Comparing Li|CSPE-10LLZTO to the CSPE-0LLZTO interface, the *R*_overall_ and *R*_interface_ of CSPE-0LLZTO were significantly higher than those for the CSPE-10LLZTO, proving that the LLZTO plays a vital role in interfacial stability. Although ionic conductivity increased from CSPE-0LLZTO to CSPE-20LLZTO, it has been challenging to demonstrate that LLZTO is a conduit for lithium ions to improve ionic conductivity. As explained above, the ionic conductivity of these CSPEs tends to follow the VTF model, where the polymer matrix is the main conduction pathway. Although the NMR technique has shown that Li ions exchange at the PEO/LLZO interface, the diffusion was extremely high, which did not contribute to the overall conductivity.^46,47^

### 3.6 Full Cell Performances

**Figure 6:**
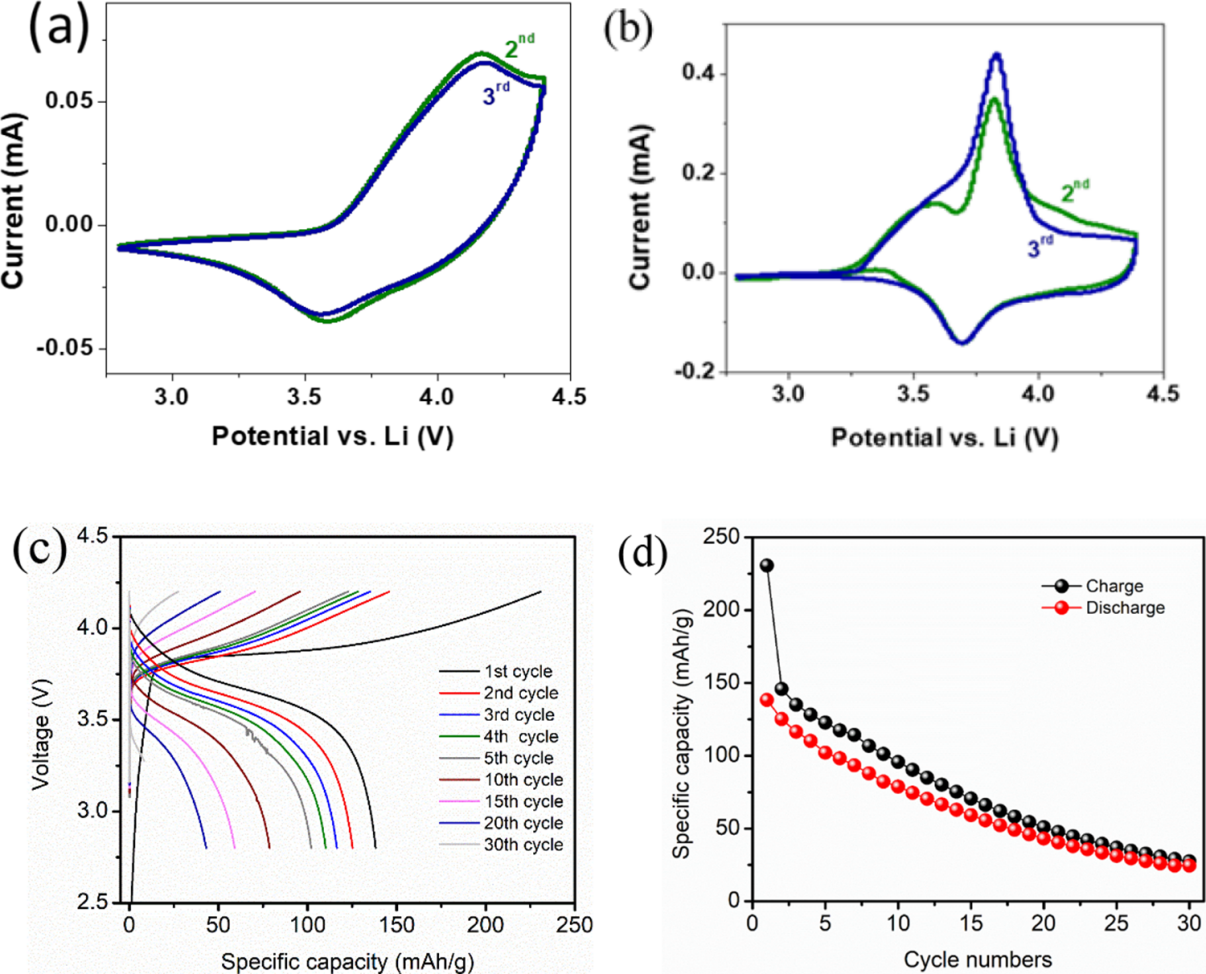
Cyclic voltammogram with a cutting-off voltage of 2.8–4.4 V at a rate of 0.2 mV/s of (a) NMC|CSPE-10LLZTO|Li and (b) NMC|Liquid electrolyte|Li; (c) charge–discharge voltage profiles at 10 mA/g at 40 °C and mass loading 1.165 mg/cm^2^; (d) capacity vs cycle number for NMC|CSPE-10LLZTO|Li.

Figure 6a shows the cyclic voltammetry of a full cell NMC|CSPE-10LLZTO|Li, which displays well-defined cathodic and anodic peaks at 3.61 and 4.24 V (versus Li^+^/Li), respectively. For comparison, Figure 6b shows the CV curves of the liquid cell. The first cycle represents the activation process of the material, and the second and third cycles showed a pair of cathodic/anodic peaks of NMC located at 3.7 and 3.87 V (versus Li^+^/Li), as expected.

All-solid-state full cell (NMC|CSPE-10LLZTO|Li) was assembled to assess the effectiveness of CSPE-10LLZTO as an electrolyte and separator with NMC as the cathode and Li metal as the anode. The active loading mass of the NMC was 1.165 mg/cm^2^. The cell was cycled at a constant current density of 10 mA/g, and the experiment was performed in the voltage range from 2.8 to 4.4 V at 40 °C. The initial charge represented the activation process of the cell. The solid-state cell delivered a discharge capacity of 140 mAh/g (Figure 6c). The specific capacity as a function of cycle number shows a loss in capacity with the cell cycle (Figure 6d). The Coulombic efficiency was approximately 80%.

### 3.7 Post-mortem Analysis

**Figure 7:**
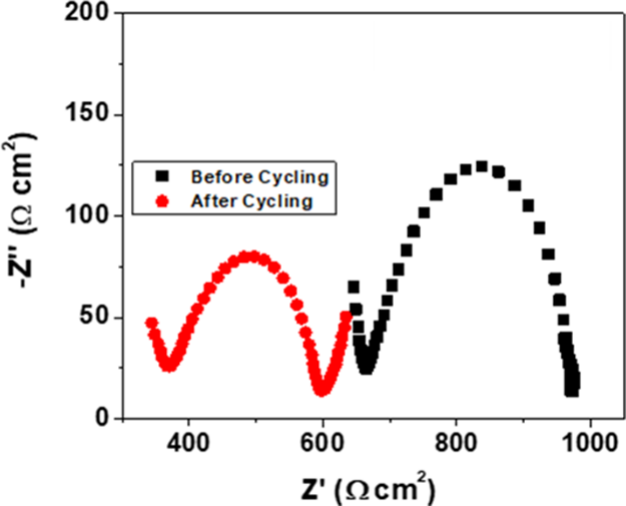
CSPE-10LLZTO before and after cycling (800 h) up to 200 μA/cm^2^ at 40 °C.

Post-mortem analysis was conducted for the CSPE-10LLZTO and CSPE-0LLZTO cells. For the CSPE-10LLZTO, Figure 7 shows that after plating and stripping for 800 h, the *R*_interface_ is reduced to ∼200 Ω cm^2^ compared to 650 Ω cm^2^ before cycling. Also, the *R*_overall_ is reduced from ∼925 to ∼600 Ω cm^2^ with no apparent changes to the shape of the impedance curves. The changes in the *R*_overall_ resistance after cycling imply an improvement in the wetting of the electrode, as can be seen in the plating and stripping. The decrease in the *R*_interface_ and *R*_ct_ with time was observed in CSPE-0LLZTO as well (Figure S6). For comparison, Figure 4c shows the EIS of CSPE-0LLZTO as well as Table 2. This phenomenon is similar to what we observed in the EIS-aged studies of all the samples at OCV at 40 °C. However, after cycling, the EIS showed lower resistance at low frequency. We believe that it is due to increased electronic conduction in the CSPE-0LLZTO cell.

The cross-sectional SEM showed intimate contact between the CSPE-10LLZTO electrolyte and the Li metal anode, as demonstrated in Figure S6. It is difficult to precisely measure the interface layer’s thickness because of the cross-section’s preparation method. Nevertheless, the thickness of the electrolyte decreased from the starting thickness of 230 μm. Also, the imperfection of the interface is apparent, as voids and gaps between the electrode and the electrolyte can be seen. Efforts to disassemble the Li|CSPE-0LLZTO|Li cell after cycling were unsuccessful because the electrolyte and Li anode could not be separated (Figure S7).

## 4 Conclusions

In this study, Li-ion-conducting solid electrolytes based on CSPE-*x*LLZTO with different concentrations of LLZTO showed excellent interfacial stability using the symmetric test. The CSPE-10LLZTO exhibits good ionic conductivity of 1.1 × 10^–4^ S/cm at 40 °C. An all-solid-state full cell, NMC|CSPE-10LLZTO|Li, has been assembled, which delivered a reversible discharge capacity of 140 mAh/g at 10 mA/g and 40 °C. Thus, cross-linking with added filler is an approach that could be used to create safe and reliable electrolytes to improve solid-state batteries. We also notice that the soft shorts play a significant role in the failure of the cells.

## Supporting Information Available

LLZTO XRD pattern; SEM picture LLZTO (400–600 nm); DSC measurements on the (a) CSPE-0LLZTO, (b) CSPE-10LLZTO, (c) CSPE-20LLZTO, and (d) CSPE-0LLZTO to study the thermal properties of the electrolyte; (e) Raman graphs of LLZTO CSPE-*x*LLZTO from 800 to 2000 cm^–1^, and (f) 720–760 cm^–1^; impedance graphs of SS|CSPE-0LLZTO|SS and SS|CSPE-20LLZTO|SS at 40 °C fitted with a simple electrical circuit model; plating and stripping of Li|CSPE-10LLZTO|Li at 40 °C and 300 μA/cm^2^; increasing the current density up to 400 μA cm^–2^ showed a slight increase in overpotential for the CSPE-10LLZTO; after cycling of Li|CSPE-0LLZTO|Li; digital images of cells after cycling and decrimping (a) CSPE-0LLZTO; (b) CSPE-10LLZTO; and (c) and the cross-section SEM between the CSPE-10LLZTO electrolyte and the Li metal anode after 800 h of cycling (PDF)
