# Supplementary material for: Cross-Linked Composite Solid Polymer Electrolyte Doped with Li6.4La3Zr1.4Ta0.6O12 for High Voltage Lithium Metal Batteries
Source: ACS Appl Mater Interfaces. 2024 Aug 19;16(34):44791–801. doi: 10.1021/acsami.4c08181 (PMC11367574; doi:10.1021/acsami.4c08181)
Supplement: Supplementary file 1 — am4c08181_si_001.pdf [file am4c08181_si_001.pdf]

Supporting information

Crosslinked Composite Solid Polymer Electrolyte Doped with $Li_{6.4}La_3Zr_{1.4}Ta_{0.6}O_{12}$ for High Voltage Lithium Metal Batteries

Lamartine Meda[1,], Kutemwa Masafwa[1], Ayssia N. Crockem[1], Jere A. Williams[1], Nila A. Beamon[1], Jada I. Adams[1], Jeremiah Tunis[1], Lingyu Yang[2], Jennifer L. Schaefer[2], James J. Wu[3]*

[1]Department of Chemistry, Xavier University of Louisiana, 1 Drexel Drive, New Orleans, LA, 70125;

[2]Deptartment of Chemical & Biomolecular Engineering, University of Notre Dame, Notre Dame, IN 46556

[3]NASA Glenn Research Center, Cleveland, Ohio 44135.

KEYWORDS: Composite solid polymer electrolytes, solid-state lithium battery, solid-state electrolyte, lithium metal anode, lithium-ion conductivity

*Corresponding Author:

Email: LMeda@Xula.edu, Tel: 504-520-5324

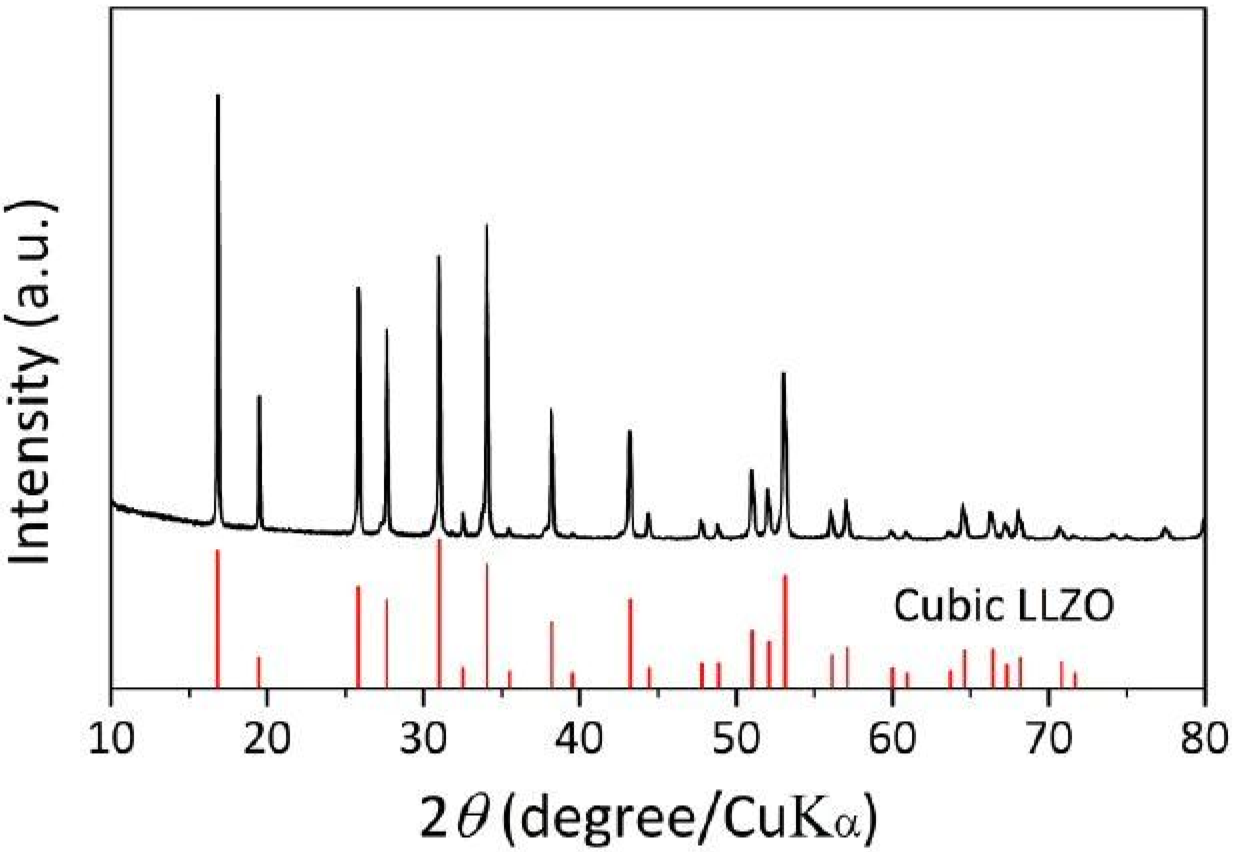

Figure S1. The LLZTO XRD pattern

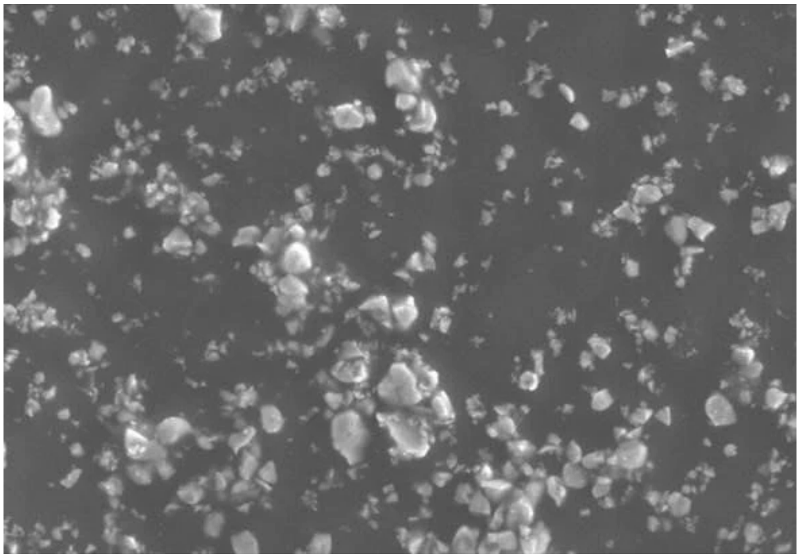

Figure S2. SEM picture of the LLZTO (400-600 nm);

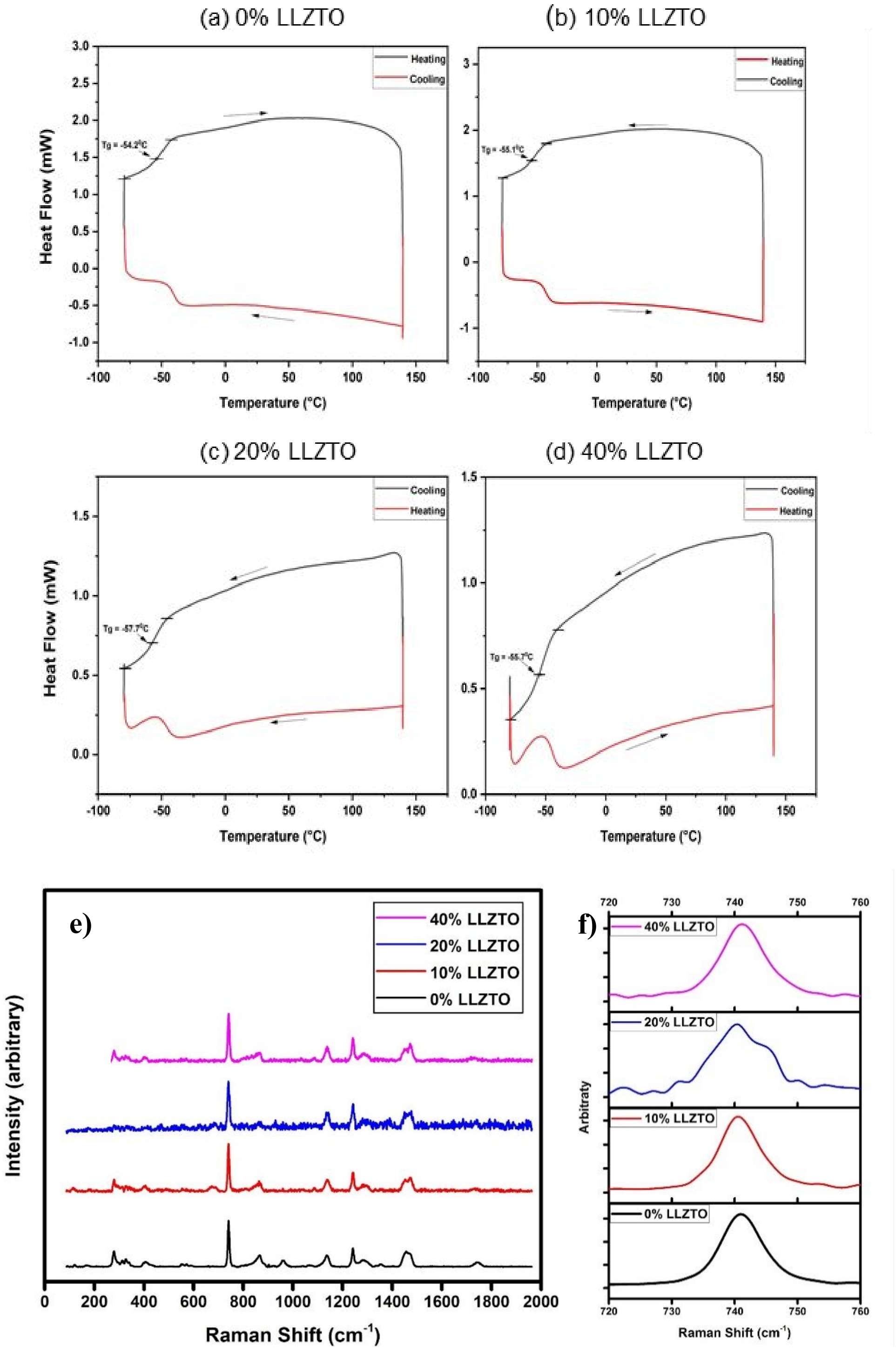

Figure S3. DSC measurements on the a) CSPE-0LLZTO, b) CSPE-10LLZTO, c) CSPE-20LLZTO, d) CSPE-0LLZTO to study the thermal properties of the electrolyte; e) Raman graphs of LLZTO CSPE-xLLZTO from 800 - 2000 cm^{-1} and f) 720 - 760 cm^{-1}.

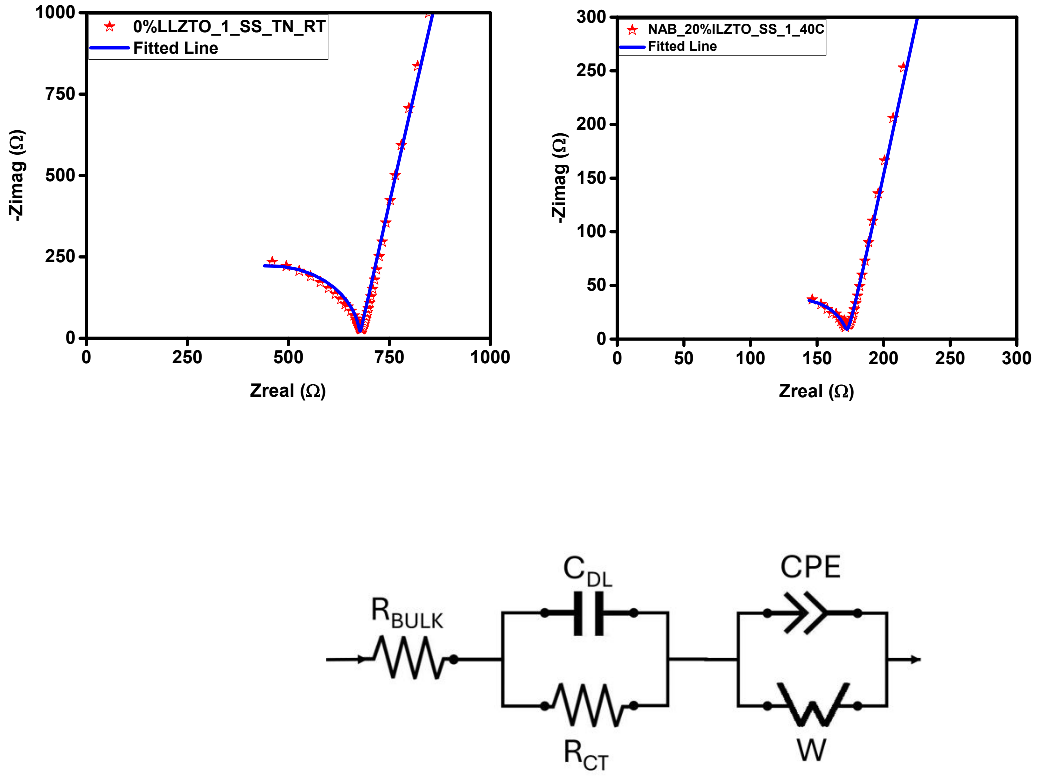

Figure S4: Impedance graphs of SS|CSPE-0LLZTO|SS and SS|CSPE-20LLZTO|SS at 40 ^{0}C fitted with a simple electrical circuit model.

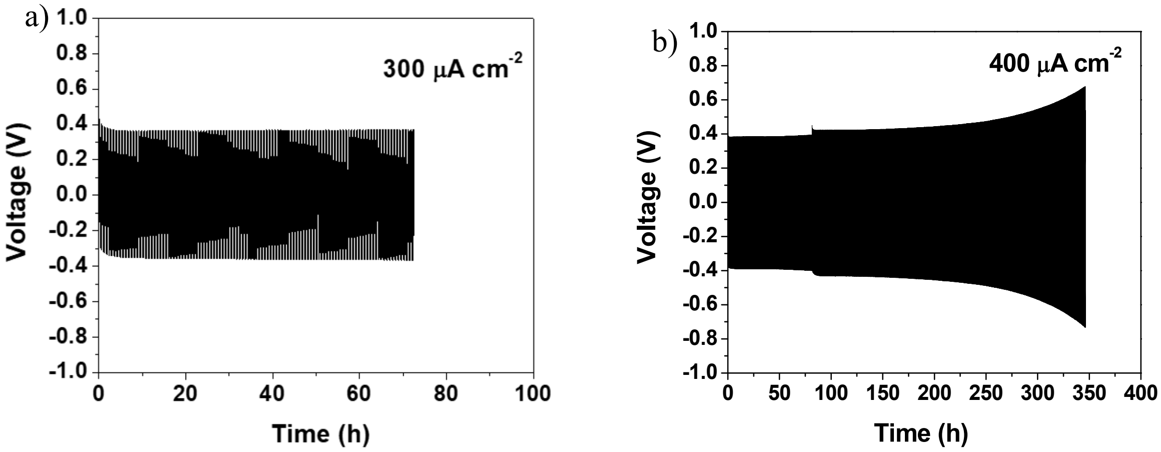

Figure S5. (a) Plating and stripping of Li|CSPE-10LLZTO|Li at 40 ºC and 300 μA/cm^2. (b) Increasing the current density up to 400 μA/cm^2 showed a slight increase in overpotential for the CSPE-10LLZTO.

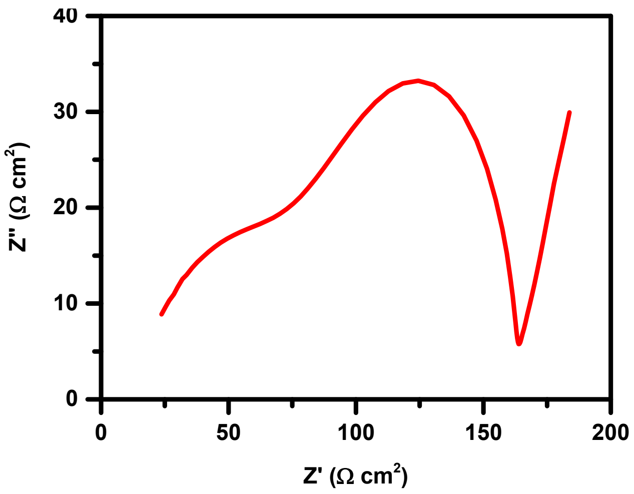

Figure S6. After cycling of Li|CSPE-0LLZTO|Li.

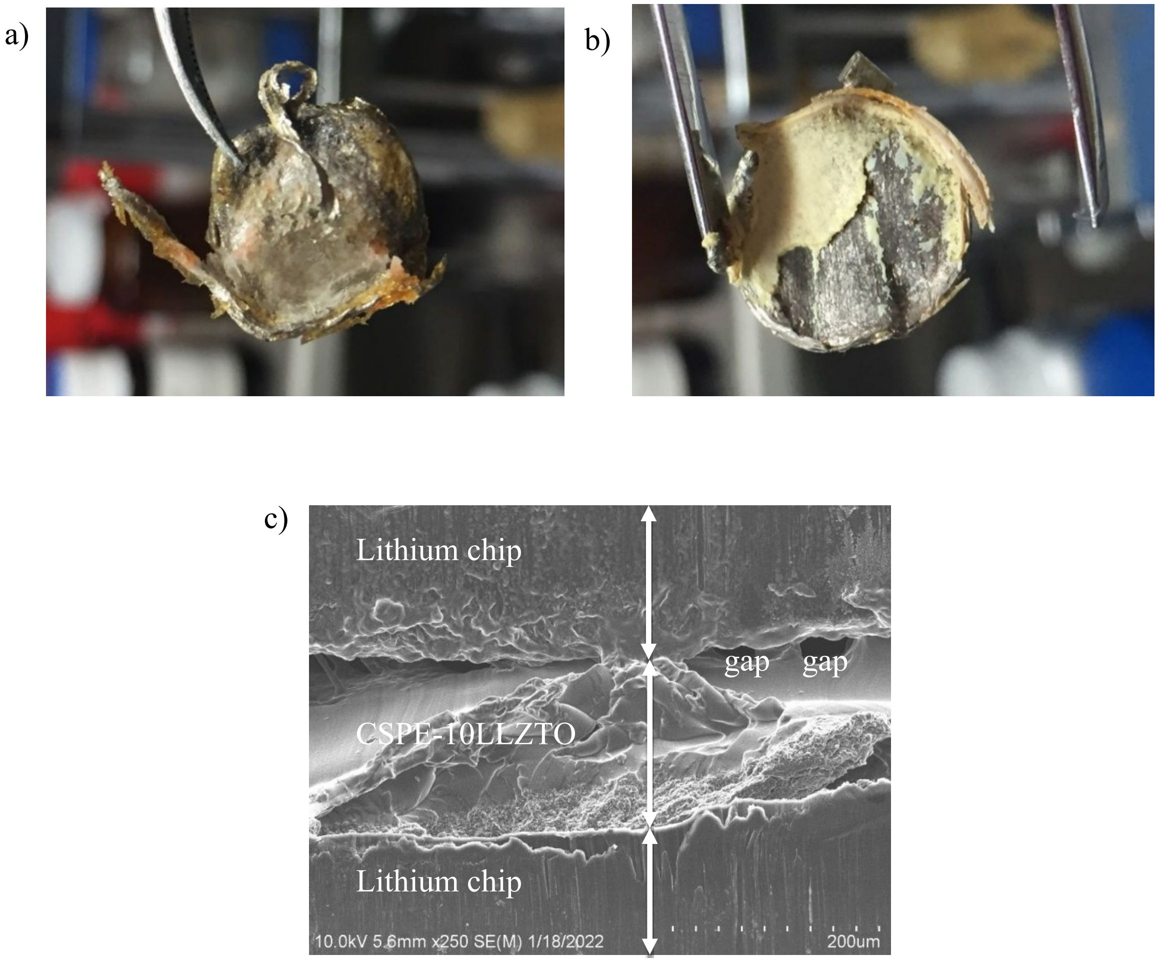

Figure S7 shows the digital images of cells after cycling and decrimping a) CSPE-0LLZTO; b) CSPE-10LLZTO; and c) the cross-section SEM between the CSPE-10LLZTO electrolyte and the Li metal anode after 800 hours of cycling.
